# Who are we? A qualitative evaluation of trainees’ perspectives on professional identity in oral and maxillofacial surgery

**DOI:** 10.1007/s40037-015-0156-1

**Published:** 2015-01-21

**Authors:** Arpan Tahim

**Affiliations:** Department of Oral and Maxillofacial Surgery, St George’s Hospital, Blackshaw Road, SW17 0QT London, UK

**Keywords:** Professional identity, Oral and maxillofacial surgery, Surgical education

## Abstract

**Introduction:**

Professional identity is becoming increasingly important in medical education in terms of developing appropriately trained and safely practising doctors. Oral and maxillofacial surgery (OMFS) is a unique surgical speciality that requires dual qualification in medicine and dentistry. Its junior trainees move between the various roles of student, doctor and dentist, and at certain times these roles may overlap. This heterogeneous early training may raise significant barriers for them in terms of understanding their professional identity and developing their own sustainable sense of belonging. This study looks to understand current trainees’ perceptions of the professional identity of an oral and maxillofacial surgeon.

**Method:**

A qualitative research methodology based on a grounded theory approach was used in this study. Data were collected using in-depth, semi-structured interviews with OMFS specialist trainees. Subsequent theories were constructed after thematic analysis.

**Results:**

A model of the professional identity of an oral and maxillofacial surgeon is proposed.

**Discussion:**

This study represents the first attempt to understand professional identity in OMFS trainees. It will provide insight into what trainees understand by the term in this speciality, as well as outlining what trainees feel are important elements to develop a sense of belonging within the speciality.

## Introduction

Professional identity has become increasingly popular as the subject for educational research. Ibarra (1999) defined it as the ‘*stable and enduring constellation of attributes, beliefs, values, motives and experiences in terms of which people define themselves in a professional role*.’[1]. It has typically been discussed in reference to vocational careers but recently it has become increasingly prominent in all forms of health care educational research including medicine, dentistry and psychology [2–5]. Understanding the holistic picture of what a professional is, rather than what they do, is of key importance for aspiring trainees as individuals in the early formative parts of their careers and for a profession as a group to help provide a context in which they can practise [6].

In the UK, oral and maxillofacial surgery (OMFS) has a unique training pathway. It requires dual qualification in both medical and dental undergraduate degrees, completion of a 2-year medical Foundation programme and a 2-year Core Surgical programme, before entry into OMFS specialist training. Traditionally trainees studied dentistry first, followed by their medical degree although the number of trainees who are entering OMFS training after studying medicine as their first degree has risen over the last 5 years. Either way, the early junior training pathway is longer than that of a typical surgical trainee. Perhaps more importantly, rather than the sequential progression of other surgical specialities—medical student, foundation trainee, core surgical trainee, specialist trainee—the early training period is highly variable as OMFS junior trainees move between undergraduate degree to junior doctor or dentist positions several times. Furthermore, this results in an extremely diverse heterogeneous junior workforce composed of both singly qualified dental graduates and singly qualified medical graduates, all of whom may or may not be considering OMFS as a future career.

With these changing roles, overlapping between student-dentist-doctor, it is not surprising that trainees may struggle with the concept of a professional identity [7]. It has further been suggested that OMFS itself is suffering from an identity crisis [8]. Research suggests a limited awareness of the scope of OMFS amongst other health care professionals and the public when compared with better-known specialities such as ENT or plastic surgery. A recent study of 100 dental patients showed that only 17 % were aware that OMFS existed [9]. A limited perception of the speciality from the outside is perhaps matched by a muddled sense of identity from within. An example of this can be demonstrated by looking at the requirement for dual qualification on a worldwide level. In the UK, the need for dual qualification has been unequivocally upheld whereas there is no such necessity internationally [10–12]. This inconsistency in the requirements may suggest a conflict in views on a professional identity for OMFS surgeons. Such a conflict may have an impact on career motivation; recent data show that approximately 40 % of students who enter their second degree with the intention of pursuing a career in OMFS, leave the speciality [13]. Although the reasons remain unclear, it is thought that the lack of a sense of belonging and identification with the speciality may contribute to their change in career choice.

It is important to understand how trainees identify with their chosen career and what they understand about being their future professional ‘self.’ Studies have suggested the importance of formation of identity alongside development of competency [14] and have sought an explicit definition of an identity within a particular profession [15, 16]. The development of identity within surgical specialities has been explored previously [17], although to date there has been limited research investigating how OMFS defines its identity.

The purpose of this study is to describe a model for professional identity in OMFS, which can be used to understand how trainees develop a sense of belonging to the profession during their lengthy early training.

## Methods

The method of data collection was the in-depth, face-to-face interview, using a semi-structured schedule, allowing for expansion of important topics where appropriate. Participants were drawn from a population of OMFS specialist trainees in the London region. Purposive sampling was used to ensure there was equal gender representation and equal representation of trainees who had studied medicine and dentistry as their first degree. The final cohort of participants in the study was thus 4 dentistry-first OMFS trainees and 3 medicine-first OMFS trainees. There were 4 males and 3 females, ranging in experience from ST3 to ST5.

Interviews were conducted off the hospital premises in a quiet location and led by the author. Interviews were transcribed, anonymized and sent to the participant for verification. Data were analyzed as they were collected, in keeping with maintaining the dynamic, iterative process important to a grounded methodology. After a period of familiarization, text segments within the data were coded to highlight important thoughts, ideas, actions or opinions. New data were continually coded, and codes were continually rejected or refined, before being clustered into categories. Meaningful categories developed into themes and subthemes, which themselves were refined and tested as further raw data were coded and integrated into this framework. As themes were further compared and consolidated, the thematic analysis led to the development of key features of professional identity.

The study was approved by the Imperial College London, Medical Education Ethics Committee.

## Results

Analysis revealed three key themes that trainees felt made up a professional identity in OMFS: i) elements that were common to all OMF surgeons, ii) factors that differentiated OMF surgeons from other medical and dental professionals and iii) ideal qualities and attributes of an OMF surgeon.

### i) Elements common to all oral and maxillofacial surgeons

#### Deep and sustained enjoyment from the speciality subject

Working in the operating theatre was a challenge that participants relished. Trainees expressed with great enthusiasm their enjoyment of performing facial surgery.

‘I love being in theatre… just ask my wife, coming back from a day in theatre and being over the moon.’ [P6]

There was also an emphasis on the breadth and variety within the speciality. This added to participants’ enjoyment while leading to a sustained interest over extended periods of time.

‘I really like the variety of the surgery we get to do. You could at one minute be taking out a tooth and the next be doing a big head and neck reconstruction… There’s just so much to choose from… it’s that variety and the skills required for doing both that make it so interesting.’ [P2]

#### Importance of facial aesthetics

Facial aesthetics was cited as an attractive feature in surgery of this nature. Participants felt performing appropriate surgical procedures should be superimposed on maintaining the aesthetics of the facial region because facial abnormalities could impact heavily on quality of life.

‘I was affected in my earlier years by the cosmetic issue surrounding [Cleft, Lip and Palate]… I could sense the societal repercussions of a cosmetic deformity as bad as that. It sincerely and deeply affected me enough to want to go into it… I would suspect that if you did an incredibly in-depth interview of most people who have entered OMFS, you would be able to identify a reason why the face was so important to them.’ [P5]

‘[the face is] a fundamental aspect of a person’s persona and their appearance, so what we do has an impact because on that region we work on, on that patient, it can really affect them. We have to do that correctly.’ [P1]

#### Regional expertise

Participants defined the speciality in terms of facial anatomy, whilst giving the ability of an OMF surgeon to manage conditions of the entire facial region a key emphasis.

‘I would say we are specialists on the face and facial skeleton… There’s no other person in the hospital who knows more about the facial skeleton and everything around it—teeth, soft tissue—than us.’ [P2]

#### Rejection of other specialities

Career selection seemed to involve rejection of other potential careers prior to the selection of OMFS. This is not surprising given the range and accessibility of other speciality options available to junior trainees in the formative stages of career selection. The process of rejection of alternative careers was heavily dependent of numerous local factors that influenced them at that time. Dental graduates usually considered entry into dental practice or other hospital dental specialities.

‘After dental school, I immersed myself in [vocational training]. For the first 6 months I had a great time but then I started getting frustrated and bored… I tried community dentistry and then some paediatric dental supervision to get a bit more experience in other dental specialities and shadowing with some orthodontists as I was toying with that idea. Then I tried OMFS.’ [P6]

Medicine-first participants consider the pursuit of careers in other head and neck specialities, predominantly ENT or plastics surgery. This could be related to a relatively greater exposure to these specialities during medical school or early postgraduate training:

‘I knew I wanted to do surgery even before graduating from medical school… I got a place on a basic surgical training rotation… I did 6 months plastic surgery during my surgical rotation and I really liked it so chose to do more plastics as I thought I wanted to specialize in it… towards the end I was seriously considering if I wanted to do it long term as I really hated [certain aspects] of it so I started looking for something else.’ [P2]

### ii) Factors that differentiate OMF surgeons from other medical and dental professionals

#### Dual qualification

Participants proudly felt dual qualification represented a trademark that no other member of the health care team possessed, although it held limited value as a standout feature within the speciality.

‘As soon as you say to people you’ve done two degrees that automatically ups you because they seem to think that’s a very impressive thing to have done. But obviously it’s just mandatory for our speciality.’ [P1]

There was also a sense that a dual qualification created a perceived boundary that prevented other specialities from expanding into regions that may be best operated upon by those with both dental and surgical skills, whilst at the same time, having a medical and a dental degree gave trainees a sense of parity with other medical and dental practitioners.

‘That’s what makes us different compared with everyone else. If that wasn’t a strict requirement, there will be other specialities trying to get their foot in. We are unique at the moment… and we should stay that way.’ [P2]

‘I’ve done full BDS, and done full MFDS, and MBBS and MRCS and can work as a dentist in practice which I still do sometimes, and I’ve done foundation and core training. That means no one can question my experience.’ [P6]

#### Expert knowledge

An extended, adaptable knowledge differentiated an OMF surgeon from dental and medical colleagues. It enabled them to maintain a broader caseload with more advanced or severe cases. Participants spoke of the extension of skills into the oral cavity:

‘We’re different because the majority of doctors have no idea even about the number of teeth we have. I mean when we receive referrals from A&E or surgery—they have no idea about what they are looking at—it’s like a mystery to them.’ [P2]

#### Technical skills

A distinct set of technical skills also helped participants define themselves. They differentiated themselves from dentists by rejecting skills that they no longer deemed applicable:

‘Things like… prosthodontics. I certainly think that whole skill set they gave of making my own dentures. Those are skills I have never ever used again.’ [P1]

When comparing themselves with medical specialities, participants focused more on added skills they have developed such as working in the confined space of the oral cavity, handling both soft and hard tissues of the face and performing both resection and reconstructive components of surgery.

‘We work in the mouth, it’s not an open area. More than other disciplines we’re working both under direct vision [rather than for example laparoscopic work] but have to do fine work in a confined space.’ [P1]

#### Supervision of inexperienced junior workforce

The majority of junior staff on OMFS teams are solely dentally qualified. It was felt their limited formal medical training influenced the role of more senior OMFS surgeons with whom they worked.

‘In other teams, registrars are less hands on for sure as their SHOs are more in tune with medicine so the majority of the stuff they can sort out on the ward. An OMFS registrar will have to sort out quite a few things on the wards as your juniors aren’t medically qualified and won’t know.’ [P2]

#### Practice within a low profile speciality

Trainees felt that OMFS did not enjoy the same status as some of its surgical counterparts amongst students, the professional workforce and the public. This is perhaps representative of the lack of a defined identity for OMFS at the moment. Trainees felt there may be problems both at an institutional level and a broader lack of awareness of the speciality:

‘[How you are perceived depends on] what the hospital thinks of your bosses and your unit there. So it is the people who make the speciality. The people in the hospital in that unit at the time.’ [P7]

‘We have a bit of an image problem in terms of how people view our profile. It’s very disparate…. People with no experience don’t really know what it is, just think it’s a cumbersome sounding speciality. They don’t know the full remit of what we do.’ [P6]

### iii) Qualities of a successful OMF surgeon

Participants spoke of particular qualities they felt OMFS consultants should possess. When questioned in detail about these skills, participants often felt such traits are not specific to OMFS consultants but are part of being a good safe practising surgeon.

Technical skills were given high priority by all participants. They stated the most important feature of a good consultant was one who was ‘*very good at their surgery*’ [P4]. Particular emphasis was placed on manual dexterity, nimble fingers and precision. Trainees felt it was their duty to their patients to master these skills:

‘You have to be good and skilled, as you are privileged. You have the privilege of treating someone else. If you can’t respect the faith the person across the table has in you then you shouldn’t be doing this job, be it surgeon, doctor, any medical professional.’ [P7]

Leadership qualities were also valued. Perhaps in keeping with its perceived low profile, trainees felt consultants should be a ‘*figure-head*’ for the speciality. Acting with ‘*assertion and confidence’*, respect from other specialities and equality with other surgeons also emerged as important in a successful OMFS consultant. In addition, trainees looked to their consultants to challenge the profession to continually improve.

The way in which a consultant acts towards others was also deemed to be a reflection of their own identity. Participants felt consultants should be approachable, inspirational and enthusiastic yet showing awareness when trainees are facing difficulties. It was also felt that consultants should play a major role in terms of ‘*reaching the speciality out to students*’. One participant recalled how during her time at medical school, she felt ‘*most nurtured by the OMFS department*’ [P5]. The importance of humility was also appreciated, and, as one participant eloquently phrased, a consultant is often judged by ‘*how they treat their equals and those below him and not how they treat the person above*.’ [P7]

Lastly, and perhaps encompassing all of the above, trainees felt that their consultants should be an example for them and other juniors to follow. The concept of a role model is well described in the literature as a powerful driver of professional identity development [18].

## Discussion

Professional identity is increasingly recognised to be important in trainee development in health care [2]. A strong sense of identity is important to provide a sense of belonging within a surgical speciality, which enables and motivates trainees while providing a context for their development [19, 20]. The importance of this study is in illustrating a model of professional identity in OMFS, which has not been described previously.

**Fig. 1:**
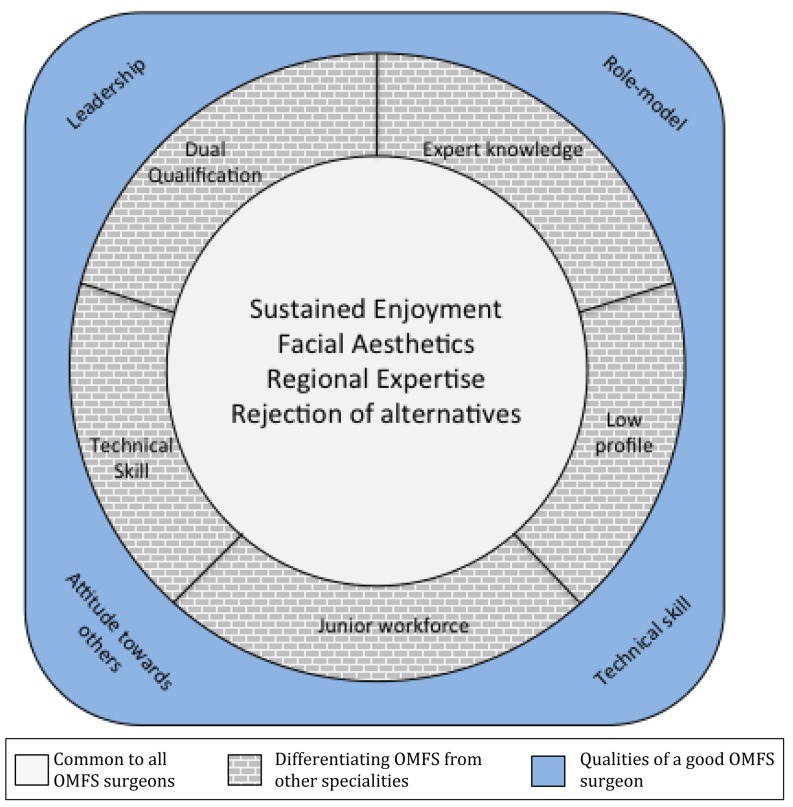
Summary of professional identity in OMFS

The results are summarized in Fig. 1. The diagram represents trainee impressions of three facets that define professional identity in OMFS: central elements common in all OMFS surgeons, which bind the speciality together from within; elements that act to distinguish OMFS from other dental and surgical specialities; with both these elements superimposed on a set of qualities and values that are important in the speciality.

OMFS early training is unusual compared with other surgical specialities. Junior trainees can be singly qualified dentists or doctors. Many may be going through the process of a second undergraduate degree. While many junior surgeons develop their sense of identity in the workplace, the complexity of OMFS early training can make this difficult. Appreciating a trainee’s understanding of their professional identity will provide insight for future junior surgeons to help them understand their roles and aims in their professional development. This will better enable them to develop their sense of belonging, which will in turn stimulate their enthusiasm and motivation in a lengthy, demanding training pathway.

There are a number of limitations in this study. By adopting a grounded theory approach, rich detailed descriptions of social phenomena have enabled conclusions of the study to evolve from the data itself. This has allowed novel theories that have not previously been described to be developed, which has been particularly useful in this study. However, the data are limited by being confined to participants’ perceptions. Also this study population was limited to the London area, thereby potentially introducing a regional bias. Furthermore the author is also pursuing a career in OMFS, which is a further source of bias in a study of this nature due to the potential for individual views and ideas to be superimposed on emerging theories. Attempts to minimize this included internal validation and reflection, along with triangulation of findings where possible. Additionally, due to the small number of OMFS surgical trainees in the region, several participants were known to the author, although participants seemed to be fully engaged and candid during the interviews.

The generalizability of this study is also important to address. Although the target audience to which these findings are directly relevant is small and highly specialized, the concept of professional identity is becoming increasingly popular and this study aims to add to this literature base. Findings could more widely relate to early professional identity development in other surgical specialities, along with development through other graduate entry undergraduate programmes.

## Conclusions

Professional identity development has been reported as a key feature in medical education. This study represents one of the first attempts to understand professional identity in OMFS trainees and is useful to help provide an understanding of what trainees understand by the term in OMFS. This study could provide the context for further research, which could look to explore the process by which trainees develop their sense of professional identity, and how its development can be integrated into early OMFS training pathways.

## Essentials

- Professional identity is becoming increasingly important in medical education in terms of developing appropriately trained and safe practising doctors.
- Early OMFS training is complicated and highly varied, leading to difficulties in identity development.
- There is limited research exploring professional identity in OMFS.
- Professional identity in OMFS is composed of three key facets.
- Development of professional identity in OMFS may represent an important component in future training.
